# Identification and Expression of *Secreted In Xylem* Pathogenicity Genes in *Fusarium oxysporum* f. sp. *pisi*

**DOI:** 10.3389/fmicb.2021.593140

**Published:** 2021-04-09

**Authors:** Sascha Jenkins, Andrew Taylor, Alison C. Jackson, Andrew D. Armitage, Helen J. Bates, Andrew Mead, Richard J. Harrison, John P. Clarkson

**Affiliations:** ^1^School of Life Sciences, Warwick Crop Centre, University of Warwick, Wellesbourne Campus, Warwick, United Kingdom; ^2^NIAB-EMR, East Malling Research, Kent, United Kingdom; ^3^Natural Resources Institute, University of Greenwich, Kent, United Kingdom; ^4^Computational and Analytical Sciences, Rothamsted Research, Harpenden, United Kingdom

**Keywords:** *Fusarium oxysporum* f. sp. *pisi*, PEA, Fusarium wilt, *formae speciales*, race, *Secreted In Xylem*, gene expression, qPCR

## Abstract

*Fusarium oxysporum* is a soilborne fungal plant pathogen responsible for causing disease in many economically important crops with “special forms” (*formae speciales*) adapted to infect specific plant hosts. *F. oxysporum* f. sp. *pisi* (FOP) is the causal agent of Fusarium wilt disease of pea. It has been reported in every country where peas are grown commercially. Disease is generally controlled using resistant cultivars possessing single major gene resistance and therefore there is a constant risk of breakdown. The main aim of this work was to characterise *F. oxysporum* isolates collected from diseased peas in the United Kingdom as well as FOP isolates obtained from other researchers representing different races through sequencing of a housekeeping gene and the presence of *Secreted In Xylem* (*SIX*) genes, which have previously been associated with pathogenicity in other *F. oxysporum* f. spp. *F. oxysporum* isolates from diseased United Kingdom pea plants possessed none or just one or two known *SIX* genes with no consistent pattern of presence/absence, leading to the conclusion that they were foot-rot causing isolates rather than FOP. In contrast, FOP isolates had different complements of *SIX* genes with all those identified as race 1 containing *SIX1*, *SIX6*, *SIX7*, *SIX9*, *SIX10*, *SIX11*, *SIX12*, and *SIX14*. FOP isolates that were identified as belonging to race 2 through testing on differential pea cultivars, contained either *SIX1*, *SIX6*, *SIX9*, *SIX13, SIX14* or *SIX1*, *SIX6, SIX13*. Significant upregulation of *SIX* genes was also observed *in planta* over the early stages of infection by different FOP races in pea roots. Race specific *SIX* gene profiling may therefore provide potential targets for molecular identification of FOP races but further research is needed to determine whether variation in complement of *SIX* genes in FOP race 2 isolates results in differences in virulence across a broader set of pea differential cultivars.

## Introduction

*Fusarium oxysporum* is the most widely dispersed and economically important plant pathogenic species in the *Fusarium* genus as it infects numerous hosts and causes extensive crop losses (Leslie and Summerell, 2006). In 2012 it was identified as fifth in a list of the top ten fungal plant pathogens in terms of scientific and economic importance, just behind *Fusarium graminearum* (Dean et al., 2012). *F. oxysporum* is responsible for a wide range of plant diseases, usually causing a vascular wilt but also causes root and bulb rots (Edel-Hermann and Lecomte, 2019). *F*. *oxysporum* is a species complex with “special forms” (*formae speciales*; f. spp.), adapted to infect specific hosts plants. These are thought to have evolved though convergent evolution and many are therefore polyphyletic (Fourie et al., 2011; van Dam et al., 2018); hence, isolates from one f. spp. may be more closely related to isolates infecting other hosts than to each other (O’Donnell et al., 1998). In *F. oxysporum*, more than 150 host specific *formae speciales* have been described (Edel-Hermann and Lecomte, 2019), including well studied and economically important examples such as *F. oxysporum* f. sp. *cubense* (banana), f. sp. *lycopersici* (tomato), f. sp. *cepae* (onion), and f. sp. *pisi* (pea). *F. oxysporum* f. sp. *lycopersici* (FOL), *cubense*, and *pisi* are also examples of f. spp. which contain multiple economically damaging races, which are specialised to infect certain cultivars of a host species; for example, f. sp. *cubense* tropical race 4 which is devasting the popular Cavendish cultivar of banana in the tropics (Ploetz, 2015).

The emergence of new pathogenic races in *F. oxysporum* f. spp. is a constant threat, driven by the evolutionary adaptation of the pathogen’s complement of effector genes, and their function, to overcome host resistance (Takken and Rep, 2010). A well-documented example of this type of evolutionary adaption between pathogen and host is that of FOL and tomato. Several FOL races have evolved to overcome I (immunity) genes in tomato that confer resistance to FOL, by loss or mutation of *Secreted In Xylem* (*SIX*) genes, which code for small cysteine rich proteins secreted into the xylem sap of tomato during infection. So far, there are 14 characterised *SIX* genes in FOL (Houterman et al., 2007; Lievens et al., 2009; Schmidt et al., 2013), and all (other than *SIX13*) are located on chromosome 14, a lineage specific (LS) chromosome found only in FOL. This is also known as a pathogenicity chromosome due to its ability to confer virulence in a non-pathogenic *F. oxysporum* isolate when artificially transferred (Ma et al., 2010; Schmidt et al., 2013). Several *SIX* genes (*SIX1*, *3*, *5*, and *6*) have been shown to be directly involved in pathogenicity of FOL in tomato, and have been shown to be essential for full virulence through gene knockout studies (Rep et al., 2004; Houterman et al., 2009; Gawehns et al., 2014). *SIX* genes have also been identified in other *F. oxysporum* f. spp. including f. sp. *cubense*, f. sp. *cepae*, f. sp. *niveum*, f. sp. *pisi*, f. sp. *Melonis*, and f. sp. *vasinfectum* (Chakrabarti et al., 2011; Meldrum et al., 2012; Covey et al., 2014; Fraser-Smith et al., 2014; Sasaki et al., 2015; Taylor et al., 2016). The presence/absence and sequence variation within *SIX* genes has been used as a new approach to distinguish between *F. oxysporum* f. spp. and even between races. For example, sequence differences in *SIX8* have been used to distinguish race 4 from races 1 and 2 in *F. oxysporum* f. sp. *cubense* (Fraser-Smith et al., 2014).

*Fusarium* wilt caused by *F. oxysporum* f. sp. *pisi* (FOP) is a major disease of pea that has been reported in every country where peas are grown commercially (Kraft, 1994). *F. oxysporum* has also been identified as a potentially important pathogen in the pea root rot complex, in addition to *Fusarium solani* and *Fusarium avenaceum* (Chittem et al., 2015). FOP can result in severe crop losses especially when peas are included in crop rotations more often than recommended, allowing inoculum levels to build up, particularly as chlamydospores can remain viable in the soil for 10 years (Kraft et al., 1994). To date, four races of FOP have been characterised: race 1, race 2, race 5, and race 6 (Haglund and Kraft, 1970, 1979), with races 1 and 2 currently the most important globally, and the only races identified in the United Kingdom (Biddle and Cattlin, 2007). Resistance to FOP by pea cultivars is conferred by single dominant genes (one for each race) for races 1, 5, and 6; however, race 2 resistance has been shown to be quantitative with a continuous scale of disease severity (McPhee et al., 2012; Bani et al., 2018). The documented method to determine FOP race is through the host response observed following inoculation of a set of pea differential cultivars. These have been used in several studies to distinguish between the four races (Haglund and Kraft, 1979; McPhee et al., 1999; Kraft and Pfleger, 2001; Bani et al., 2012). Cultivars are assessed for wilt symptoms, which begin with the downward curling then progressive yellowing and drying of the leaves from the base of the stem to the apex of the plant and as the disease develops (Kraft, 1994).

Although *SIX* genes have been identified in FOP previously, this was usually as part of studies focusing on other f. spp. One study identified *SIX1*, *SIX13*, and *SIX14* in FOP and suggested that they could contribute to pathogenicity (Fraser-Smith et al., 2014). Additional *SIX* genes were identified in three isolates of FOP representing races 1, 2, and 5 as part of a larger screening of *SIX* gene presence in *F. oxysporum* f. sp. *cepae* (FOC). Here, up to six *SIX* genes were present (*SIX7*, *SIX10*, *SIX11*, *SIX12*, *SIX13*, and *SIX14*) with different complements across the three putative FOP races tested (Taylor et al., 2016). Furthermore, a recent genome study of a FOP race 5 isolate identified four *SIX* genes; *SIX1*, *SIX9*, *SIX13*, and *SIX14* (Williams et al., 2016). However, none of these studies determined the expression of these *SIX* genes *in planta*, which is an important indicator of whether they may have a role in facilitating infection.

The main aim of this study was to determine the complement of *SIX* genes in FOP isolates from different putative races to establish if this approach could be used to distinguish between them and provide targets for molecular diagnostics. *SIX* gene expression was then examined using quantitative polymerase chain reaction (qPCR) to determine whether *SIX* genes were expressed in the early stages of pea infection. Finally, FOP isolate race was determined using a pathogenicity test on pea differential cultivars.

## Materials and Methods

### *Fusarium* Isolate Collection

*Fusarium* isolates were obtained from the Processors and Growers Research Organisation (PGRO; crop clinic isolations and field sampling 2012–2014) and also from sampling of United Kingdom pea plants displaying symptoms of root rot or wilt, from the major pea growing areas of Yorkshire, Lincolnshire, and Suffolk in 2015/2016 (Supplementary Table 1). Isolations were made from plant root and stem sections which were surface sterilised in a 10% bleach/sterile water solution (v/v) for 3 min, then washed twice in sterile water to remove bleach residue. Three sections from the sterilised material were plated onto potato dextrose agar (39 g L^–1^ PDA; Merck, United Kingdom) containing 2 mL L^–1^ chlortetracycline [10 mg mL^–1^ in 1/1 methanol/water (v/v)] and incubated at 20°C for 5–7 days. *Fusarium* cultures distinct in morphology were sub-cultured onto fresh PDA plates grown for approx. 7 days at 20°C. In addition, 28 isolates of FOP were obtained from other researchers mostly from outside the United Kingdom (mainly the United States) who had pre-assigned a putative race based on their own tests using sets of differential pea cultivars or based on infection of certain cultivars in the field (Supplementary Table 2). These were sub-cultured onto PDA and grown for 5–7 days at 20°C. Spore suspensions of all isolates were made in potato dextrose broth (PDB) + 20% glycerol (v/v) for storage on ceramic beads at −80°C.

### Molecular Characterisation of *F. oxysporum* Isolates

The identity of *Fusarium* isolates collected from United Kingdom fields and the additional FOP isolates with a preassigned putative race was first confirmed by sequencing part of the *translation elongation factor 1α* (*TEF*) gene. Three 5 mm agar plugs were removed from the growing edge of each actively growing culture and placed in 20 mL of 50% PDB in a 50 mL centrifuge tube. Tubes were incubated at 20°C for 5 days positioned at a 45° angle. The mycelium of each isolate was then removed from the PDB by centrifugation (3,000 rpm for 15 min) and rinsed twice with sterile water (centrifugation at 3,000 rpm for 15 min each time) before it was lyophilised.

DNA was extracted from freeze-dried mycelium using a DNeasy plant mini kit (Qiagen, Hilden, Germany), in accordance with manufacturer’s protocol, with a minor modification whereby the mycelium was first homogenised in a lysing matrix-A tube (MP Biomedicals, CA, United States) in a FastPrep-24^TM^ machine (MP Biomedicals, United Kingdom) set at 6 ms^–1^ for 40 s. Identification of *Fusarium* isolates was carried out by polymerase chain reaction (PCR) amplification of part of the *TEF* gene using published primers (Supplementary Table 3). All PCR reactions were set up using REDTaq^®^ ReadyMix^®^ (Sigma-Aldrich, United Kingdom) in 20 μL reaction volumes containing 1 μL of DNA and a final concentration of 0.5 μM of each primer. Thermocycling conditions were: one cycle of 2 min at 94°C; 30 cycles of 45 s at 94°C, 30 s at 64°C and 1 min at 72°C, followed by one cycle of 5 min at 72°C. PCR amplicons were visualised using gel electrophoresis (1% agarose gel containing GelRed^TM^ at 2 μL per 100 mL of gel), purified using the QIAquick PCR Purification Kit (Qiagen) and sequenced using the forward primer sequence (GATC, Germany). Sequences were subjected to basic local alignment search tool (BLAST searches; Boratyn et al., 2013) using the National Centre of Biotechnology Information (NCBI) database to identify species based on sequence identity values.

**FIGURE 1:**
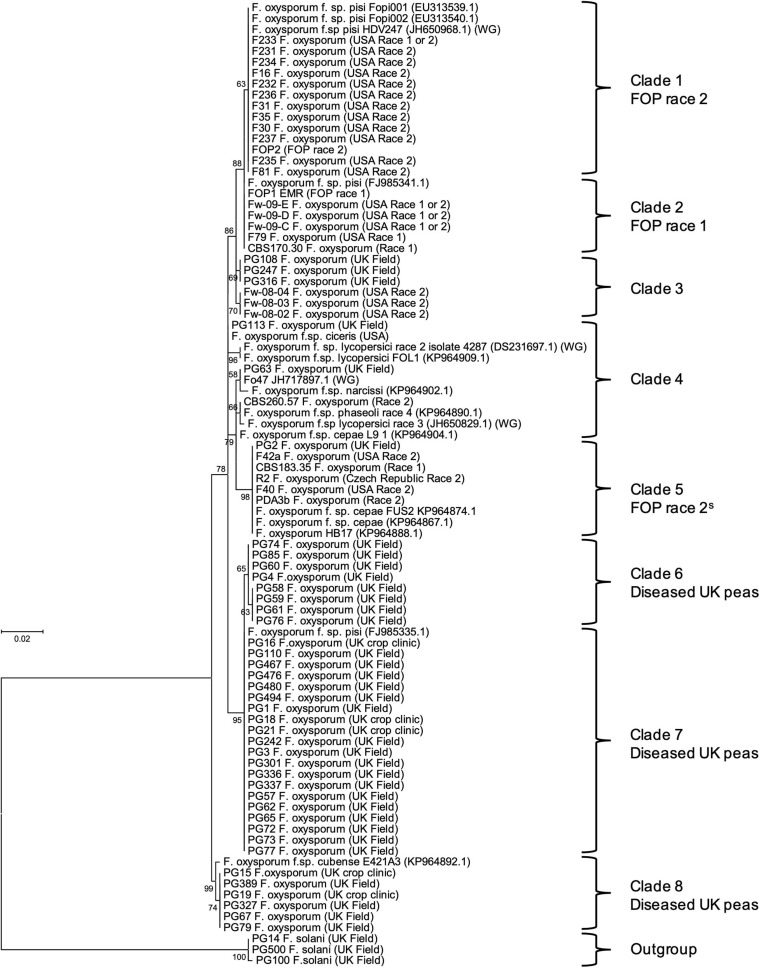
Maximum likelihood tree for 40 *Fusarium oxysporum* isolates from diseased United Kingdom peas (coded PG), 28 *F. oxysporum* f. sp. *pisi* (FOP) isolates from other researchers (with original race designation) and other *F. oxysporum formae speciales*, based on an alignment of *translation elongation factor 1α* (*TEF*) sequences. Numbers represent bootstrap percentage values from 1,000 replicates. Scale bar indicates 0.02 substitutions per site. The tree is rooted through three *F. solani* isolates (outgroup).

*Translation elongation factor 1α* sequences were aligned and trimmed in Molecular Evolutionary Genetics Analysis version 7.0 (MEGA7; Kumar et al., 2016) and used to construct a maximum-likelihood tree using a Kimura 2-parameter model (Kimura, 1980) as determined using the “Find best DNA/Proteins model (ML)” tool in the software. Sequences of other *F. oxysporum* f. spp. isolates from Taylor et al. (2016) and NCBI database were also included for reference (Genbank numbers in Figure 1). Bootstrap values were inferred from 1,000 replicates (Felsenstein, 1985) and percentages displayed next to the relevant branch.

### Identification of *SIX* Genes in *F. oxysporum* Isolates From Pea

In order to develop primers for *SIX* gene identification, whole genome sequencing of three pathogenic isolates of FOP (FOP1 EMR, F81, and R2) provisionally race typed by other researchers as putative race 1 (FOP1 EMR) and putative race 2 (F81 and R2), was conducted using both Illumina MiSeq and Oxford Nanopore Technology (ONT). DNA was extracted from freeze-dried mycelium using the Macherey-Nagel Nucleospin Plant II kit (Thermo Fisher Scientific, United Kingdom). For Illumina library preparation DNA was sheared in a Covaris M220 using microTUBE-50 (Covaris, United Kingdom) and size selected using the Blue Pippin (Sage Science, United Kingdom). Illumina libraries were constructed with a PCR-free method using NEBNext End Repair, NEBNext dA-tailing and Blunt T/A ligase (New England Biolabs, United Kingdom) and Illumina TruSeq barcodes. Libraries were sequenced on an Illumina MiSeq using MiSeq Reagent kit V3 (2 × 300 bp PE). For ONT library preparation, DNA was sheared using a g-TUBE (Covaris, United Kingdom) and then size selected for fragments greater than 10 kb using the Blue Pippin. Libraries were constructed using the ligation sequencing kit with NEBNext enzymes as detailed in the ONT protocol and run using a R9.5 FLO-MIN107 flow cell on the GridION.

Nanopore reads were assembled using SMARTdenovo (Ruan, 2016) and corrected using Racon for 10 rounds (Vaser et al., 2017) and Nanopolish (Loman et al., 2015), before being polished using Illumina MiSeq reads of the same isolates in Pilon (Walker et al., 2014). Quast was used to summarise assembly statistics and BUSCO v3 was used to assess completeness of gene space within the assembly (Gurevich et al., 2013; Simão et al., 2015). RepeatModeler and transposonPSI were used to identify repetitive and low complexity regions (Haas, 2007; Smit et al., 2013–2015).

Unmasked assembled genome sequences were imported into Geneious (v. 6.1.5) and used to conduct BLAST (Boratyn et al., 2013) searches for homologs of the 14 *SIX* genes previously identified in FOL (sequences obtained from NCBI). Sequences of positive hits (plus 500 bp up and downstream) were aligned in MEGA7 and used to check the utility of previously published primers for each of the 14 *SIX* genes (Lievens et al., 2009; Taylor et al., 2016). Where these primers did not match target sequences in FOP, new primers were designed using Primer3Plus (Untergasser et al., 2007) and checked for self-hybridisation potential using Eurofins Oligo Analysis Tool (Eurofins, 2016). These optimised primers were then used to determine the presence of *SIX* genes in 40 *F. oxysporum* isolates from United Kingdom peas and the 28 FOP isolates obtained from other researchers with previously assigned race type. PCR reactions for each *SIX* gene were set up using REDTaq^®^ ReadyMix^®^ (Sigma-Aldrich, United Kingdom) in 20 μL reaction volumes with 1 μL of DNA and 1 μL each of 10 μM primers with thermocycling conditions as follows: one cycle for 2 min at 94°C; 30 cycles of 45 s at 94°C, 30 s at primer annealing temperature (Supplementary Table 3) and 1 min at 72°C, followed by one cycle of 7 min at 72°C. PCR products were visualised using gel electrophoresis (1% agarose gel containing GelRed^TM^ at 2 μL per 100 mL of gel).

*Secreted In Xylem* gene sequences were also aligned with homologs from other *F. oxysporum* f. sp. in MEGA7 and used to construct maximum-likelihood trees as described above. The nucleotide substitution models used are listed in Supplementary Table 4. Additional *SIX* sequences were downloaded from NCBI as described above.

### Expression of *SIX* Genes *in Planta*

An *in vitro* method where peas were grown and inoculated with FOP isolates on an agar medium was adapted from Taylor et al. (2016). Autoclaved ATS medium [1 M KNO_3_, 1 M KPO_4_, 1 M MgSO_4_, 1 M Ca(NO_3_)_2_, 20 mM Fe-EDTA, 70 mM H_3_BO_3_, 14 mM MnCl_2_, 0.5 mM CuSO_4_, 1 mM ZnSO_4_, 0.2 mM Na_2_MoO_4_, 10 mM NaCl, 0.01 mM CoCl_2_, and 0.45% Gelrite (Duchefa Biochemie, Haarlem, Netherlands)] was used to three-quarter fill square petri dishes (12 × 12 × 1.7 cm, Greiner Bio-One, United Kingdom) and once set, the top 4.5 cm of the gel was removed with a sterile spatula. Pea seeds (cv. Little Marvel, susceptible to all FOP races) were sterilised in a 10% bleach/sterile water (v/v) solution for 5 min, then rinsed with sterile distilled water (SDW) until no bubbles remained. Six pea seeds were spaced evenly across the plate and pushed into the cut edge of the agar before the lid was replaced and secured with tape. Stacks of 6–8 plates were wrapped in cling film and incubated at 20°C for 3 days in the dark, and then for a further 5 days in light/dark (16 h day length). Conidial suspensions of FOP1 EMR (putative race 1), F81 (putative race 2), and R2 (putative race 2 from a different *TEF* clade) were made by releasing spores from 2-weeks-old agar plates with 10 mL SDW and filtering through three layers of miracloth. Spore suspensions were adjusted to 1 × 10^6^ spores mL^–1^ using SDW with the addition of 200 μL of Tween20 L^–1^ and 1 mL pipetted directly onto pea roots and spread by tilting the plate. Plates were re-sealed and returned to the 20°C (16 h photoperiod) incubator in the arrangement of a split pot design. Pea root samples were taken at 10 time points (8, 16, 24, 36, 48, 72, and 96 hours post inoculation), including a 0 h time point (pre-inoculation) and an uninoculated control (SDW/Tween only) collected at 96 hpi. Four plates per FOP isolate were sampled at each timepoint where the roots of four pea plants per plate were removed, rinsed in SDW, flash frozen in liquid nitrogen and stored at −80°C until use.

Roots were ground to a fine powder using a pestle and mortar filled with liquid nitrogen and approximately 100 mg of tissue transferred to a 2 mL tube. Frozen root material was ground further using a Dremel drill (model 398, with a rounded drill bit) and then RNA extracted using Trizol^®^ reagent (Ambion, Thermo Fisher Scientific) following the manufacturer’s guidelines. Extracted RNA was precipitated using 900 μL of lithium chloride to 100 μL RNA (250 μL LiCl_2_ + 650 μL DEPC treated water) and any DNA was removed from samples using DNase 1 (Sigma-Aldrich, United Kingdom). RNA samples were visualised on a 2% agarose gel (containing GelRed^TM^ at 2 μL per 100 mL of gel) with the addition of loading dye (Orange G, Sigma-Aldrich, United Kingdom) to check for degradation. First strand cDNA was synthesised using Superscript II reverse transcriptase (Invitrogen, Thermo Fisher Scientific, United Kingdom) following the manufacturer’s protocol.

To quantify expression of *SIX* genes, Real-Time RT-PCR was performed using the cDNA from each FOP isolate and a Roche Lightcycler 480 using the Lightcycler 480 SYBR Green 1 Master mix (Roche, United Kingdom), following the manufacturer’s protocol. Published primers (Taylor et al., 2016) were used for *SIX7*, *SIX9*, *SIX10*, *SIX12*, and *SIX13* with the remainder (*SIX1*, *SIX6*, *SIX11*, and *SIX14*) designed in this study as described above (Supplementary Table 3). Due to the multi-copy nature of *SIX1* (identified through genome sequencing) and some sequence differences between isolates, primers were designed to match one copy (copy 1, Supplementary Figure 1) of this gene in the putative race 2 isolates F81 and R2 only, and therefore no data was collected for the expression of these genes in the putative race 1 isolate FOP1 EMR. Similarly, primers were only designed for the copy of *SIX6* (Supplementary Figure 1) present in all isolates. In addition to *SIX* genes, qPCR was also conducted for *TEF* using published primers (Taylor et al., 2016; Supplementary Table 3) to determine background expression levels. All primers were used at a final concentration of 0.4 μM (except qSIX11 and qSIX14 which were used at 0.2 μM and qSIX6 which was used at 0.15 μM), using the following conditions: one cycle of 95°C for 5 min, 45 cycles of 95°C for 10 s, primer annealing temp (Supplementary Table 3) for 10 s and finally 72°C for 10 s. A melt curve analysis was used to confirm the presence of a single PCR product. Standard curves were plotted for each *SIX* gene target by using serially diluted genomic DNA and the concentration expressed relative to *TEF*. All samples were run in triplicate, for each of the four replicate root samples for each time point per FOP isolate. Relative concentration values for each *SIX* gene were log*_*e*_* transformed and analysed using Analysis of variance (ANOVA) in Genstat^®^ (release 18.1, VSN international Ltd., United Kingdom) considering the split plot blocking structure in the incubator. Significant differences between individual time points or isolates were compared with the least significant difference (LSD) at the 5% level (Supplementary Table 5).

### Race Typing of FOP Isolates

Pea differential cultivars (Table 2) were inoculated using the root dipping method adapted from Bani et al. (2012) to determine the race of FOP isolates selected from different clades of the *TEF* phylogenetic tree and with different *SIX* gene profiles. Cultures of FOP1 EMR and F79 (putative race 1), FOP2 and F81 (putative race 2), and R2 and F40 (putative variant of race 2 based on variations in *SIX* gene complement) were initiated from glycerol bead stocks on PDA and grown for 14 days at 20°C. Meanwhile, pea seeds of differential cultivars Darkskin Perfection (DSP), Mini, Sundance II, and Little Marvel (Table 2) obtained from the Germplasm Resources Unit (GRU) at John Innes Centre (Norfolk, United Kingdom) were sown in 24 cell modular trays containing vermiculite (1–3 mm diameter) until 2–3 nodes tall (approx. 14 days), in the glasshouse (25°C day, 18°C night, 16 h photoperiod). Conidial suspensions of each FOP isolate were prepared as described above and adjusted to 1 × 10^6^ spores mL^–1^ in sterile water. Pea plants from each of the differentials were removed from the vermiculite, roots washed briefly and trimmed by a third in length, before being immersed for 5 min in a spore suspension of each FOP isolate. Roots of uninoculated control plants were immersed in SDW only. Following inoculation, pea plants were transplanted into individual pots (7 × 7 × 8 cm) containing vermiculite and maintained in the glasshouse (conditions as above) where they were watered daily (or when needed) and supplemented with fertiliser (Vitax 2:1:4, diluted 100-fold). Plants were arranged in a randomised complete block design with each of the 14 blocks containing one plant of each pea cultivar. Plants were monitored for wilt symptoms each week for 41 days, commencing 7 days after inoculation, by recording the proportion of leaves showing symptoms of wilt for each plant. The number of wilted leaves at 41 dpi was calculated as a proportion of the total number and transformed using a logit transformation with an offset of one. The transformed data was analysed by ANOVA in Genstat^®^ (release 18.1, VSN international Ltd.), considering the randomised block design in the glasshouse. Treatment means from significant ANOVA’s were compared to the control and each other using the LSD at the 5% level (Supplementary Table 6).

## Results

### Molecular Characterisation of *F. oxysporum* Isolates

*Fusarium* isolates collected from diseased peas from United Kingdom fields were identified using *TEF* gene sequencing, with the majority being confirmed as *F. oxysporum*, in addition to isolates of *F. solani* and *Fusarium redolens*. A selection (40 isolates) of those identified as *F. oxysporum* were used for phylogenetic analysis, along with 28 isolates previously identified as FOP by other researchers.

The maximum likelihood phylogenetic tree based on *TEF* gene sequences, separated the *F. oxysporum* isolates from United Kingdom peas, FOP isolates and sequences from other f. spp. into a total of eight clades (based on bootstrap values > 60%; Figure 1). The majority of *F. oxysporum* isolates from diseased United Kingdom peas (coded PG) were typically found in three closely related clades (6, 7, and 8; Figure 1) and were distinct from the 28 FOP isolates from other researchers (clades 1, 2, and 5; Figure 1). In most cases, isolates putatively identified as FOP race 1 and race 2 grouped into separate but closely related clades in the tree (clade 1 for race 2 and clade 2 for race 1), supporting their putative race assignment. However, a small group of putative race 2 isolates were found in another clade (clade 5; Figure 1), alongside two isolates of FOC, suggesting a different origin or potentially even a different race type, and were designated a subset or variant of race 2 (2^S^). Generally, the 28 FOP isolates grouped separately from isolates of other *F. oxysporum* f. spp. obtained from NCBI, although other published FOP isolates grouped with FOP race 1 and 2 isolates from this study. The clear distinction of the isolates from diseased United Kingdom peas from previously race typed FOP isolates led us to hypothesise that they were of a different genetic origin.

### Identification of *SIX* Genes in *F. oxysporum* Isolates From Pea

Genome sequence analysis revealed that FOP1 EMR (race 1), F81 (race 2), and R2 (race 2) contained homologs of eight (*SIX1*, *SIX6*, *SIX7*, *SIX9*, *SIX10*, *SIX11*, *SIX12*, and *SIX14*), five (*SIX1*, *SIX6*, *SIX9*, *SIX13*, and *SIX14*), and three (*SIX1*, *SIX6*, and *SIX13*) *SIX* genes, respectively. An additional copy of *SIX6* (*SIX6*^2^) was also identified in FOP1 EMR, while F81 and R2 contained one and two additional copies of *SIX1*, respectively.

Primers optimised for FOP were used to identify *SIX* genes within the 40 *F. oxysporum* isolates from diseased United Kingdom peas and the 28 previously race typed FOP isolates. Isolates previously assigned to either FOP race 1 or 2 within *TEF* clades 2 and 1, respectively (Figure 1) contained the same complements of *SIX* genes as identified through genome sequencing for isolates FOP1 EMR (race 1, Group A) and F81 (race 2, Group B), respectively (Table 1). Similarly, the subset of putative race 2 isolates from *TEF* clade 5 (Figure 1) contained the same complement of *SIX* genes as isolate R2 (race 2, Group C, Table 1). These latter race 2 isolates from *TEF* clade 5 differed from those in *TEF* clade 1 due to the absence of *SIX9* and *SIX14*. A number of previously identified FOP isolates from other *TEF* clades (Figure 1) with a putative assignment of race 1 or 2 also contained few or no *SIX* genes (Group D, Table 1). In contrast, *F. oxysporum* isolates from diseased United Kingdom peas (coded PG) contained either one (*SIX6* or *SIX14*) or no *SIX* genes (Group E, Table 1), suggesting that they may not be wilt causing FOP isolates.

**TABLE 1:** Presence and absence of *Secreted In Xylem* (*SIX*) genes found in *Fusarium oxysporum* isolates from diseased United Kingdom peas and in *F. oxysporum* f. sp. *pisi* (FOP) isolates from other researchers (with previous putative race designation) as determined by PCR.

Group	Race*	Isolate	Location	Clade (*TEF*)	*SIX* gene presence/absence										Race (*SIX*)
					1	6	6^2^	7	9	10	11	12	13	14	
A	1	FOP1 EMR	United Kingdom	Clade 2	+	+	+	+	+	+	+	+	−	+	1
	1	F79	United States		+	+	+	+	+	+	+	+	−	+	1
	1	CBS170.30	United States		+	+	+	+	+	+	+	+	−	+	1
	1/2	Fw-09-C	United States		+	+	+	+	+	+	+	+	−	+	1
	1/2	Fw-09-D	United States		+	+	+	+	+	+	+	+	−	+	1
B	2	F81	United States	Clade 1	+	+	−	−	+	−	−	−	+	+	2
	2	FOP2	United Kingdom		+	+	−	−	+	−	−	−	+	+	2
	2	F231	United States		+	+	−	−	+	−	−	−	+	+	2
	2	F31	United States		+	+	−	−	+	−	−	−	+	+	2
	2	F234	United States		+	+	−	−	+	−	−	−	+	+	2
	2	F235	United States		+	+	−	−	+	−	−	−	+	+	2
	2	F16	United States		+	+	−	−	+	−	−	−	+	+	2
	2	F232	United States		+	+	−	−	+	−	−	−	+	+	2
	2	F236	United States		+	+	−	−	+	−	−	−	+	+	2
	2	F35	United States		+	+	−	−	+	−	−	−	+	+	2
	2	F30	United States		+	+	−	−	+	−	−	−	+	+	2
	2	F237	United States		+	+	−	−	+	−	−	−	+	+	2
	2	F233	United States		+	+	−	−	+	−	−	−	+	+	2
C	2	R2	Czechia	Clade 5	+	+	−	−	−	−	−	−	+	−	2^S^
	2	F40	United States		+	+	−	−	−	−	−	−	+	−	2^S^
	2	PDA3b	United States		+	+	−	−	−	−	−	−	+	−	2^S^
	2	F42a	United States		+	+	−	−	−	−	−	−	F	−	2^S^
D	2	Fw-08-04	United States	Clade 3	+	+	−	−	−	−	−	−	−	−	RR
	1	CBS183.35	Unknown	Clade 5	+	F	−	−	−	−	−	−	−	+	RR
	2	CBS260.57	Unknown	Clade 4	−	−	−	−	−	−	−	−	−	−	RR
	2	Fw-08-02	United States	Clade 3	+	+	−	−	−	−	−	−	−	−	RR
	2	Fw-08-03	United States	Clade 3	+	+	−	−	−	−	−	−	−	−	RR
	1/2	Fw-09-E	United States	Clade 2	−	F	−	−	+	−	−	−	−	+	RR
E		PG1	United Kingdom Field	Clade 7	−	−	−	−	−	−	−	−	−	−	RR
		PG2	United Kingdom Field	Clade 5	−	−	−	−	−	−	−	−	−	−	RR
		PG3	United Kingdom Field	Clade 7	−	+	−	−	−	−	−	−	−	−	RR
		PG4	United Kingdom Field	Clade 6	−	−	−	−	−	−	−	−	−	−	RR
		PG15	United Kingdom	Clade 8	−	+	−	−	−	−	−	−	−	−	RR
		PG16	United Kingdom	Clade 7	−	−	−	−	−	−	−	−	−	−	RR
		PG18	United Kingdom	Clade 7	−	−	−	−	−	−	−	−	−	−	RR
		PG19	United Kingdom	Clade 8	−	+	−	−	−	−	−	−	−	−	RR
		PG21	United Kingdom	Clade 7	−	−	−	−	−	−	−	−	−	−	RR
		PG57	United Kingdom Field	Clade 7	−	−	−	−	−	−	−	−	−	−	RR
		PG58	United Kingdom Field	Clade 6	−	−	−	−	−	−	−	−	−	+	RR
		PG59	United Kingdom Field	Clade 6	−	−	−	−	−	−	−	−	−	+	RR
		PG60	United Kingdom Field	Clade 6	−	−	−	−	−	−	−	−	−	−	RR
		PG61	United Kingdom Field	Clade 6	−	−	−	−	−	−	−	−	−	+	RR
		PG62	United Kingdom Field	Clade 7	−	−	−	−	−	−	−	−	−	−	RR
		PG63	United Kingdom Field	Clade 4	−	−	−	−	−	−	−	−	−	−	RR
		PG65	United Kingdom Field	Clade 7	−	−	−	−	−	−	−	−	−	−	RR
		PG67	United Kingdom Field	Clade 8	−	−	+	−	−	−	−	−	−	−	RR
		PG72	United Kingdom Field	Clade 7	−	−	−	−	−	−	−	−	−	−	RR
		PG73	United Kingdom Field	Clade 7	−	−	−	−	−	−	−	−	−	−	RR
		PG74	United Kingdom Field	Clade 6	−	−	−	−	−	−	−	−	−	−	RR
		PG76	United Kingdom Field	Clade 6	−	−	−	−	−	−	−	−	−	+	RR
		PG77	United Kingdom Field	Clade 7	−	−	−	−	−	−	−	−	−	−	RR
		PG79	United Kingdom Trial	Clade 8	−	+	−	−	−	−	−	−	−	−	RR
		PG85	United Kingdom Field	Clade 6	−	−	−	−	−	−	−	−	−	−	RR
		PG108	United Kingdom Field	Clade 3	−	−	−	−	−	−	−	−	−	−	RR
		PG110	United Kingdom Field	Clade 7	−	−	−	−	−	−	−	−	−	−	RR
		PG113	United Kingdom Field	Clade 4	−	−	−	−	−	−	−	−	−	−	RR
		PG242	United Kingdom Field	Clade 7	−	−	−	−	−	−	−	−	−	−	RR
		PG247	United Kingdom Field	Clade 3	−	−	−	−	−	−	−	−	−	+	RR
		PG301	United Kingdom Field	Clade 7	−	+	−	−	−	−	−	−	−	−	RR
		PG316	United Kingdom Field	Clade 3	−	−	−	−	−	−	−	−	−	−	RR
		PG327	United Kingdom Field	Clade 8	−	+	−	−	−	−	−	−	−	−	RR
		PG336	United Kingdom Field	Clade 7	−	+	−	−	−	−	−	−	−	−	RR
		PG337	United Kingdom Field	Clade 7	−	+	−	−	−	−	−	−	−	−	RR
		PG389	United Kingdom Field	Clade 8	−	+	−	−	−	−	−	−	−	−	RR
		PG467	United Kingdom Field	Clade 7	−	−	−	−	−	−	−	−	−	−	RR
		PG476	United Kingdom Field	Clade 7	−	−	−	−	−	−	−	−	−	−	RR
		PG480	United Kingdom Field	Clade 7	−	−	−	−	−	−	−	−	−	−	RR
		PG494	United Kingdom Field	Clade 7	−	−	−	−	−	−	−	−	−	−	RR

Isolates are grouped by *SIX* gene profile. +, Band (gene) present; F, faint band present; −, no band (gene) present; RR, root rot causing isolates; 2^*S*^, represents a subset of FOP race 2. * Denotes putative race designation before identification of *SIX* gene complement.

*Secreted In Xylem* gene sequences in FOP were also compared with *SIX* gene homologs in other *F. oxysporum* f. spp. Similar copies of *SIX1* (copies 1 and 2) in F81 and R2 grouped together (Supplementary Figure 1), whereas the third copy in R2 was similar to the sequence from *F. oxysporum* f. sp. *medicaginis* (*Medicago truncatula*). The additional copy of *SIX6* (*SIX6*^2^) in FOP1 EMR grouped closer to the sequence identified in *F. oxysporum* f. sp. *cubense*, than to the copies of *SIX6* found in all races (Table 1). The sequence for *SIX13* in FOP was very similar to the sequence in *F. oxysporum* f. sp. *narcissi*, with isolates from both being grouped into the same clade (Supplementary Figure 1). Generally, the sequences of *SIX* genes in FOP differed from those identified in other *F. oxysporum* f. spp., with most resulting in distinct branches in the phylogenetic trees (Supplementary Figure 1).

### Expression of *SIX* Genes *in Planta*

**FIGURE 2:**
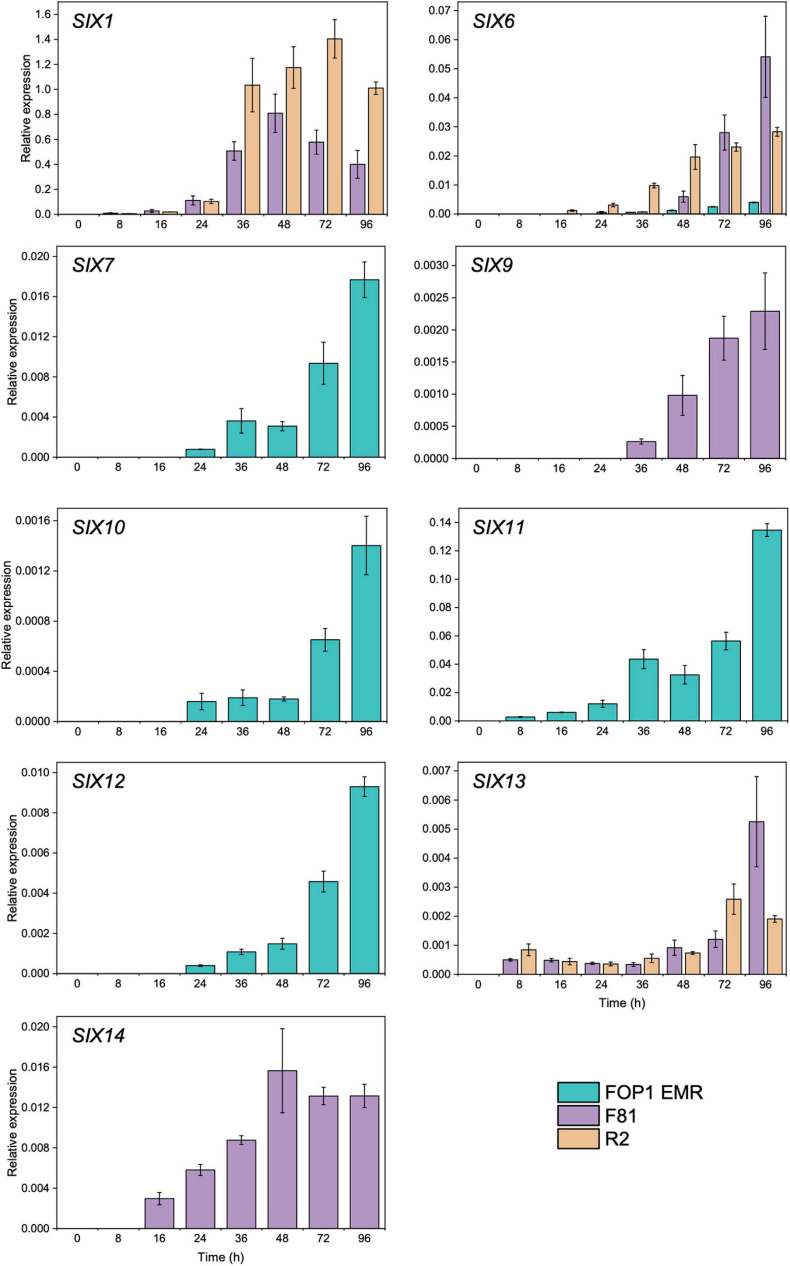
Expression of *Secreted In Xylem* (*SIX*) genes in pea roots infected with FOP isolates FOP1 EMR (race 1), F81 (race 2), and R2 (race 2^*S*^) as determined by reverse transcription qPCR of RNA. Expression was calculated relative to the *TEF* and the mean value displayed [error bars represent Standard error of the mean (SEM)].

Of the *SIX* genes identified in FOP1 EMR (*SIX1*, *SIX6*, *SIX7*, *SIX9*, *SIX10*, *SIX11*, *SIX12*, and *SIX14*), F81 (*SIX1*, *SIX6*, *SIX9*, *SIX13*, and *SIX14*), and R2 (*SIX1*, *SIX6*, and *SIX13*), the vast majority of those tested (*SIX1* in FOP1 EMR was not tested) were expressed *in planta*, across the early stages of infection (8–96 hpi), following inoculation of pea seedlings (Figure 2). *SIX7*, *SIX10*, *SIX11*, and *SIX12* expression levels (present in FOP1 EMR only) increased at every time point over the experiment, with significant increases observed from 72 hpi onward (Figure 2 and Supplementary Table 5). *SIX6* was expressed in all three isolates (Figure 2), although levels were significantly less in FOP1 EMR compared with the other isolates F81 and R2 (Supplementary Table 5). Although *SIX14* and *SIX9* were both present in FOP1 EMR and F81, these genes were only expressed in F81, with expression levels increasing over time during the early stages of infection (Figure 2). Expression of *SIX13* increased over time in both F81 and R2, with similar levels in both isolates, until 96 hpi where expression in F81 was significantly higher than in R2 (Supplementary Table 5).

Due to the multiple copies of *SIX1*, only FOP isolates F81 and R2 were included in the analysis for this gene, as the sequence of *SIX1* in FOP1 EMR varied from the copies in F81 and R2. Expression of *SIX1* increased over time in both isolates, with levels peaking at 48 hpi for F81 and at 72 hpi for R2 (Figure 2). However, the expression levels for *SIX1* in F81 were significantly lower than in R2 at 36, 72, and 96 hpi (Supplementary Table 5). No *SIX* gene expression was detected for uninoculated control pea collected at 96 hpi.

### Race Typing of FOP Isolates

**FIGURE 3:**
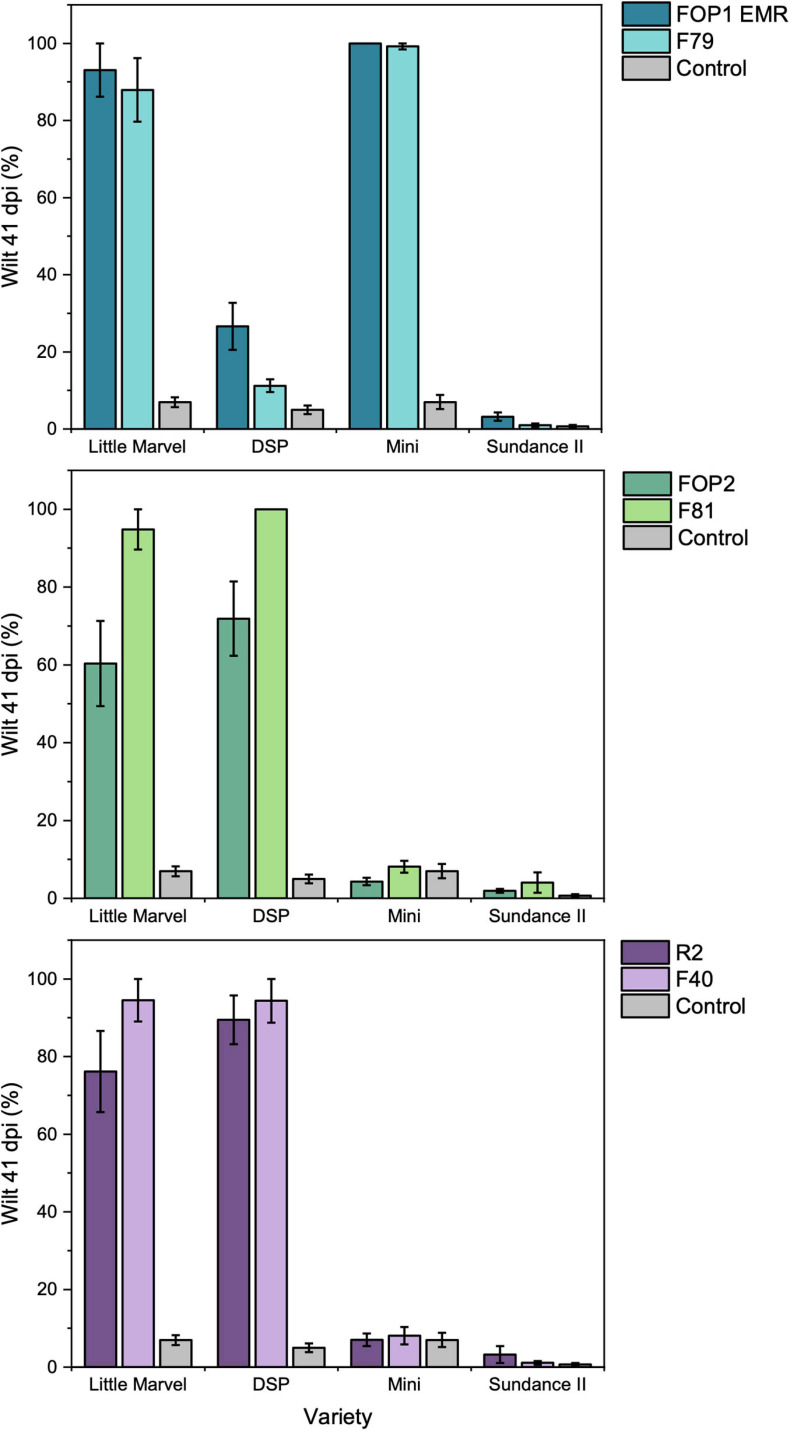
Pathogenicity of *SIX* FOP isolates on four differential cultivars of pea (Little Marvel, DSP, Mini, and Sundance II) using the root dip inoculation method. Top = putative race 1 isolates; middle = putative race 2 isolates and bottom = putative race 2^*S*^ isolates. Values are average percentage wilt of 14 inoculated plants, error bars are SEM.

In the root dip inoculation test there was a significant interaction observed between the six FOP isolates (FOP1 EMR, F79, FOP2, F81, R2, and F40) and the four pea cultivars (Little Marvel, DSP, Mini, and Sundance II) tested (*p* < 0.001; Supplementary Table 6). All *F. oxysporum* isolates were highly pathogenic on the universal susceptible cultivar Little Marvel, each causing over 60% leaf wilt by 41 dpi (Figure 3). Cultivar DSP (resistant to FOP race 1 and susceptible to race 2) was resistant to FOP1 EMR and F79, with less than 25% of leaves wilted, and highly susceptible to FOP2, F81, R2, and F40, with more than 81% of leaves wilted confirming these isolates as race 1 and race 2, respectively (Figure 3). This race designation was further confirmed by results on cultivar Mini which was highly susceptible to FOP1 EMR and F79 and resistant to FOP2, F81, R2, and F40. Finally, the resistance of cultivar Sundance II to FOP1 EMR and F79 confirmed these isolates as FOP race 1 (Figure 3 and Table 2). However, unexpectedly, isolates FOP2, F81, R2, and F40 caused no significant wilt compared to the control for Sundance II which is reported to be susceptible to FOP race 2.

**TABLE 2:** Summary of expected (Exp.) and observed (Obs.) results for the resistance and susceptibility of pea differential cultivars to different FOP isolates representing preliminary races 1, 2, and 2^*S*^ as designated based on *SIX* gene complement.

Race	Isolate	Pea cultivar							
		Little Marvel		DSP		Mini		Sundance II	
		Exp.	Obs.	Exp.	Obs.	Exp.	Obs.	Exp.	Obs.
1	FOP1 EMR	S	S	R	R	S	S	R	R
	F79	S	S	R	R	S	S	R	R
2	FOP2	S	S	S	S	R	R	S	R
	F81	S	S	S	S	R	R	S	R
2^*S*^	R2	S	S	S	S	R	R	S	R
	F40	S	S	S	S	R	R	S	R

*Pea cultivars were inoculated by dipping seedling roots in spore suspensions. S, susceptible; R, resistant reactions.*

## Discussion

In this study, we have identified for the first time, the presence of nine *SIX* genes in FOP, with distinct complements of genes occurring in races 1 (*SIX1*, *SIX6*, *SIX7*, *SIX9*, *SIX10*, *SIX11*, *SIX12*, and *SIX14*) and 2 (*SIX1*, *SIX6*, *SIX9*, *SIX13, SIX14* or *SIX1*, *SIX6, SIX13*). All *SIX* genes tested were expressed *in planta* (except *SIX9* and *SIX14* in race 1), suggesting they could be functionally involved in the early stages of FOP infection. Race testing of FOP isolates using the pea differential cultivars confirmed the difference between the isolates putatively designated as race 1 or race 2. Therefore, the unique *SIX* gene complement identified in FOP race 1 isolates could be used as a way to distinguish these from race 2. Additionally, the complete absence or the presence of just one or two *SIX* genes in *F. oxysporum* isolates from diseased United Kingdom pea plants, suggests that they are not FOP isolates and preliminary tests (Jenkins, 2018) indicated that they cause foot and root rot rather than wilt in peas.

In previous research where just three FOP isolates were included in a study to identify complements of *SIX* genes in multiple isolates of FOC and a selection of other *F. oxysporum* f. spp., *SIX7*, *SIX10*, *SIX11*, *SIX12*, and *SIX14* were identified in FOP race 1, *SIX13* and *SIX14* were identified in race 2 and *SIX13* identified in race 5 leading to the conclusion that there was variation *SIX* gene complement between different races (Taylor et al., 2016). However, FOP race identity was not confirmed on plants and the presence of *SIX* genes relied on primers designed to *SIX* gene sequences from FOL and FOC. It is frequently documented that sequences for *SIX* genes vary across *F. oxysporum* f. spp. (Lievens et al., 2009; van Dam et al., 2016), which could account for the discrepancy in results between this and the present study where *SIX* gene primers were optimised for FOP. In this current study, nine *SIX* genes were identified in the whole genome sequences of three FOP isolates (Jenkins, 2018), which enabled previously published primers to be evaluated for their complementarity against FOP specific *SIX* gene sequences, and where necessary, new FOP specific primers were designed.

The variation of *SIX* gene complement in isolates identified previously by other researchers as race 2 was unexpected; the majority of these isolates contained five *SIX* genes (*SIX1, SIX6, SIX9, SIX13*, and *SIX14*) while the remainder lacked *SIX9* and *SIX14*. These two groups of race 2 isolates also separated into different *TEF* clades suggesting different origins. One hypothesis is that these two groups could actually represent different races. Although FOP race 5 isolates were not represented in this study, recent genome analysis of one isolate has identified the presence of *SIX1*, *SIX9*, *SIX13*, and *SIX14* (Williams et al., 2016), which is very close to the *SIX* gene profile of the majority of our race 2 isolates, lacking just *SIX6*. Although this further supports the idea that profiling *SIX* genes or other putative effector genes may be informative for distinguishing some races in FOP, the variation in *SIX* gene presence within race 2 isolates needs to be understood. Despite these differences, representative isolates from both groups of race 2 isolates resulted in the same reactions in the small set of differential pea cultivars tested here and were therefore confirmed as FOP race 2. This was based on expected reactions observed for all these isolates on the cultivars Little Marvel (susceptible), DSP (resistant), and Mini (resistant). Unexpectedly, however, these isolates resulted in a resistant reaction for cv. Sundance II where the expected outcome for race 2 isolates is susceptibility. There is no clear explanation for this although variation in FOP race 2 isolates has been documented previously and races originally identified as “old” race 3 and 4 have now been combined under race 2 despite some differences in pea differential response (Kraft and Haglund, 1978; Kraft, 1994). For instance, the reaction of the differential pea cultivar New Season has been reported to vary with different isolates of race 2 (Kraft, 1994; Kraft and Pfleger, 2001). To properly resolve the races in FOP and potentially separate the two groups of race 2 isolates with different *SIX* gene profiles, further tests are required using an improved and expanded set of pea differential cultivars. The addition of RNAseq data, as well as *SIX* gene profiling, might also identify presence/absence of other potential effectors which could help to explain the differences in these two groups of race 2 isolates more definitively.

Differences in *SIX* gene profile between *F. oxysporum* f. spp. races as observed in this study for FOP has been reported previously, and in cases where these have been identified as avirulence (*Avr*) genes, this has arisen as a means of evading detection by corresponding host R genes. For instance, *SIX4* (*Avr 1*) which is present in FOL race 1 isolates is absent in races 2 and 3 preventing host recognition by the tomato *I* and *I-1* resistance alleles (Takken and Rep, 2010). *SIX4* is not required for general virulence, rather it suppresses the ability of other resistance alleles (*I−2* and *I−3*) to confer resistance. In a different strategy to overcome plant resistance, single point mutation variants in *SIX3* (*Avr2*) in FOL race 3 prevents recognition by the tomato *I−2* allele (Takken and Rep, 2010). Similarly, *SIX8* gene sequence can be used to distinguish race 4 from races 1 and 2 in *F. oxysporum* f. sp. *cubense* (Fraser-Smith et al., 2014). It could be hypothesised therefore, that the loss of non-essential *SIX* genes from FOP race 1 (*SIX7*, *10*, *11*, and *12*) in race 2, and the gain of *SIX13*, could have contributed to the ability of race 2 to overcome resistant pea cultivars. Similarly, the loss of *SIX6* in FOP race 5 (Williams et al., 2016) which is present in FOP races 1 and 2, could suggest it is non-essential for FOP virulence and may have allowed race 5 to evade host detection. In contrast, the presence of *SIX1* in all FOP races could imply it is essential for virulence, although this would require testing with gene knockout studies.

The majority of *F. oxysporum* isolates obtained from diseased pea plants from United Kingdom fields contained no *SIX* genes, with only some isolates containing one *SIX* gene (*SIX6* or *SIX14*). In the *TEF* phylogenetic tree, the majority of these isolates (with and without *SIX* genes) also grouped into separate clades from the FOP isolates suggesting a different origin and were shown in unpublished studies to cause foot and root rot (which results in similar symptoms to FOP in mature pea plants in the field) rather than wilt caused by FOP isolates (Jenkins, 2018). The general lack of *SIX* genes in foot and root-rotting *F. oxysporum* isolates suggests a different mode of infection to FOP resulting in slightly different disease symptoms. *SIX* genes (*SIX1*, *SIX6*, *SIX8*, *SIX9*, *SIX11*, and *SIX14*) have previously been identified in naturally occurring *F. oxysporum* isolates in Australia, which provided evidence of horizontal gene transfer (HGT) from f. spp. of an agricultural origin into the natural isolate population, especially in the case of *SIX1* and *SIX6* (Rocha et al., 2016). It was suspected that the *SIX* genes were non-functional in the natural population due to their low frequency (Rocha et al., 2016), and this could also be the case for the root rot causing *F. oxysporum* isolates in pea.

This current study showed that of the *SIX* genes identified in different races of FOP (*SIX1*, *SIX6*, *SIX7*, *SIX9*, *SIX10*, *SIX11*, *SIX12, SIX13*, and *SIX14*), all of those tested were expressed (except *SIX9* and *SIX14* in race 1) during the early stages of pea infection, hence suggesting a role in virulence and providing targets for future functional studies. Expression of *SIX* genes in other *F. oxysporum* f. spp. has been examined previously; for instance, a similar study in FOC confirmed expression *in planta* of all seven *SIX* genes present (*SIX3*, *SIX5*, *SIX7*, *SIX9*, *SIX10*, *SIX12*, and *SIX14*) following inoculation of onion seedling roots (Taylor et al., 2016).

*Secreted In Xylem 1* gene expression was observed in FOP race 2 isolates F81 and R2 but was not confirmed for race 1 isolate FOP1 EMR due to variation in the sequence. However, additional RNAseq analysis (Jenkins, 2018) showed that *SIX1* was expressed in FOP1 EMR at 96 hpi. *SIX1* sequences have been shown to vary between races of *F. oxysporum* f. sp. *cubense*, with the sequence of *SIX1* in sub-tropical race 4 differing from the sequence found in race 1, 2, and tropical race 4 (Meldrum et al., 2012). *SIX1* has previously been shown to be essential for virulence in FOL (Rep et al., 2005), *F. oxysporum* f. sp. *conglutinans* (Li et al., 2016) and *F. oxysporum* f. sp. *cubense* tropical race 4 (Widinugraheni et al., 2018).

*Secreted In Xylem 6* has also been shown to be expressed in the early stages of FOL infection on tomato seedlings (Gawehns et al., 2014) and was also found to be expressed from 3 dpi in watermelon roots infected with *F. oxysporum* f. sp. *niveum* (Niu et al., 2016). *SIX6* has also been shown to be necessary for full virulence in FOL (Gawehns et al., 2014). Only one copy of *SIX6* was used for qPCR analysis due to the similarities in sequences between the two copies in FOP1 EMR. Additional RNAseq analysis (Jenkins, 2018) showed that there was no expression for *SIX6*^2^ in FOP1 EMR, suggesting this gene is non-functional, although tests would be needed to confirm this.

Although *SIX9* and *SIX14* were present in the genome of FOP1 EMR, and were detected via PCR, they were not highly expressed *in planta*, yielding expression values lower than the limit of detection, suggesting they could be pseudogenes and therefore not important in infection. However, *SIX14* was highly expressed in FOP race 2 isolates where it was present. Both *SIX9* and *SIX14* were expressed during infection of onion seedlings with FOC in a similar study (Taylor et al., 2016). *SIX9* was identified in the 20 highest expressed genes of a pathogenic FOC isolate during infection, whereas *SIX14* was expressed at much lower levels and was ranked at 3,277th (Armitage et al., 2018). Interestingly, *SIX9* was also identified in a non-pathogenic *F. oxysporum* isolate from onion (Armitage et al., 2018), suggesting it may not be directly related to virulence. This was also shown in *F. oxysporum* f. sp. *radicis-cucumerinum* where disruptions in *SIX9* did not significantly affect virulence (van Dam et al., 2017).

Further research is now required to understand the role of *SIX* genes in FOP pathogenicity through knockout studies as implemented in *F. oxysporum* f. spp. (Rep et al., 2005; Houterman et al., 2009; Gawehns et al., 2014). Additionally, proteomic studies could also help determine the presence of functional proteins in the sap of pea plants (Houterman et al., 2007; Lievens et al., 2009).

Overall, our findings provide a greater understanding of *SIX* genes in FOP and their presence and absence in different races. This provides a foundation for a better understanding of the evolution of virulence in FOP races and also potentially improve the diagnosis and control of FOP in pea in the future, as the current method of differential testing to race type FOP isolates is not always reliable. Using effectors to understand plant–pathogen gene interactions could also lead to more robust plant resistance to FOP, which is less likely to breakdown with the evolution of new races.

## Data Availability Statement

Oxford Nanopore and Illumina reads for the three sequenced isolates (FOP1 EMR, F81, and R2) were submitted to the NCBI Sequence Read Archive with accessions SRX8613878–SRX8613883 and are available under bioproject PRJNA641642.

## Author Contributions

SJ planned and performed the experiments, analysed the data, created the figures, and drafted and edited the manuscript. AT and AJ helped to plan experiments and analyse data. HB carried out the sequencing of FOP genomes while AA carried out the genomic analyses. AM created the experimental designs and provided statistical analyses. RH obtained funding, planned experiments, and edited the manuscript. JC obtained funding, planned experiments, and wrote and edited the manuscript. All authors contributed to the article and approved the submitted version.

## Conflict of Interest

The authors declare that the research was conducted in the absence of any commercial or financial relationships that could be construed as a potential conflict of interest.
