# Supplementary material for: Identification and Expression of Secreted In Xylem Pathogenicity Genes in Fusarium oxysporum f. sp. pisi
Source: Front Microbiol. 2021 Apr 9;12:593140. doi: 10.3389/fmicb.2021.593140 (PMC8062729; doi:10.3389/fmicb.2021.593140)
Supplement: Supplementary file 1 [file Data_Sheet_1.PDF]

Table S1 *Fusarium oxysporum* isolates (PG1-PG85) obtained from the Processors and Growers Research Organisation (PGRO) and collected from diseased peas from UK fields (PG108- PG494) sampled during the 2015 and 2016 growing seasons. Isolates used for identification using *translation elongation factor 1α* (*TEF*) gene sequencing and *Secreted In Xylem* (*SIX*) gene screening.

Isolate	Country of origin	Location	Source	Sampling year	Isolated from	Pea Cultivar
PG1	UK	Shropshire	Shropshire	-		
PG2	UK	Shropshire	Shropshire	-		
PG3	UK	Shropshire	Shropshire	-		
PG4	UK	Shropshire	Shropshire	-		
PG15	UK	East Anglia	Crop clinic 2012	2012		
PG16	UK	East Anglia	Crop clinic 2012	2012		
PG18	UK	East Anglia	Crop clinic 2012	2012		
PG19	UK	East Anglia	Crop clinic 2012	2012		
PG21	UK	East Anglia	Crop clinic 2012	2012		
PG57	UK	East Anglia	A14/26	2014		
PG58	UK	East Anglia	A14/27	2014		
PG59	UK	East Anglia	A14/27	2014		
PG60	UK	Northhants	A14/43	2014		
PG61	UK	Northhants	A14/43	2014		
PG62	UK	Northhants	A14/43	2014		
PG63	UK	Warwickshire	A14/37	2014		
PG65	UK	Northhants	A14/38	2014		
PG67	UK	Yorkshire	A14/45	2014		
PG72	UK	Oxfordshire	A14/51	2014		
PG73	UK	Oxfordshire	A14/51	2014		
PG74	UK	Oxfordshire	A14/51	2014		
PG76	UK	Oxfordshire	A14/51	2014		
PG77	UK	Oxfordshire	A14/51	2014		
PG79	UK	Unknown	Garlic and Awais trial	2014		
PG85	UK	Worcestershire	A14/36	2014		
PG108	UK	Yorkshire	Field 116	2015	Root	Oasis
PG110	UK	Yorkshire	Field 116	2015	Root	Oasis
PG113	UK	Yorkshire	Field 133	2015	Root	Oasis
PG242	UK	Suffolk	Field 461 4A	2015	Root	Naches
PG247	UK	Suffolk	Field 461 8B	2015	Root	Naches
PG301	UK	Suffolk	Field 451	2015	Root	Terrain
PG316	UK	Suffolk	Field 461 11B	2015	Root	Naches
PG327	UK	Suffolk	Field 461 13A	2015	Root	Naches
PG336	UK	Yorkshire	BE1	2015	Root	Kite
PG337	UK	Yorkshire	BE1	2015	Root	Kite
PG389	UK	Suffolk	Field 461 12B	2015	Root	Naches
PG467	UK	Yorkshire	Field 98	2016	Stem	Ashton
PG476	UK	Lincolnshire	OAS71	2016	Stem	Oasis
PG480	UK	Yorkshire	Field 97	2016	Stem	Ashton
PG494	UK	Yorkshire	Field 99	2016	Stem	Ashton

Table S2 Putative *Fusarium oxysporum* f. sp. *pisi* (FOP) isolates from the UK and overseas with original isolate race type from the sender. Identification was carried out based on sequencing of the *translation elongation factor 1α* (*TEF*) gene and a new race designated by *TEF* phylogeny and presence/absence of *Secreted In Xylem (SIX*) genes.

Isolate name	Original FOP race	Country of origin	Contact name	Race (*TEF/SIX*)
CBS183.35	1	CBS culture collection	CBS	1
CBS170.30	1	CBS culture collection	CBS	1
CBS260.51	2	CBS culture collection	CBS	1
PDA3b	2	USA	Dr R. J McGee	2^S
F79	1	USA	Dr R. J McGee	1
Fw-09-E	1 or 2	WA, USA	Dr L. Porter	RR
F236	2	MN, USA	Dr L. Porter	2
F232	2	MN, USA	Dr L. Porter	2
F30	2	OR, USA	Dr L. Porter	2
F81	2	WA, USA	Dr L. Porter	2
Fw-08-04	2	WA, USA	Dr L. Porter	RR
F42a	2	WA, USA	Dr L. Porter	2^S
Fw-08-02	2	WA, USA	Dr L. Porter	RR
F16	2	OR, USA	Dr L. Porter	2
F35	2	OR, USA	Dr L. Porter	2
F31	2	OR, USA	Dr L. Porter	2
Fw-08-03	2	WA, USA	Dr L. Porter	RR
F235	2	MN, USA	Dr L. Porter	2
F234	2	MN, USA	Dr L. Porter	2
F231	2	MN, USA	Dr L. Porter	2
F40	2	WA, USA	Dr L. Porter	2^S
F233	2	MN, USA	Dr L. Porter	2
F237	2	OR, USA	Dr L. Porter	2
Fw-09-D	1 or 2	WA, USA	Dr L. Porter	1
Fw-09-C	1 or 2	WA, USA	Dr L. Porter	1
R2	2	Czech Republic (via PGRO)	Dr L. Herold (PGRO)	2^S
FOP2	2	UK (historic Warwick HRI isolate)	Dr C. Linfield	2
FOP1 EMR	1	UK	NIAB-EMR	1

Table S3 Primer pairs used in the characterisation of *Fusarium* species, *SIX* gene presence/absence and *SIX* gene expression studies, with primer name, sequence, annealing temperature and relevant publications included.

Gene	Primer	Sequence 5'-3' (forward/reverse)	Annealing temp. (˚C)	Reference
TEF	exTEF-F/ FUexTEF-R	ACCCGGTTCAAGCATCCGATCTGCGA/ AGCTTGCCRGACTTGATCTCACGCTC	64	Taylor et al. (2016)
SIX1	SIX1 ALL F1/R1	CTCGGCACCCTCTCAATC/ CATTGGTGACAGCATCGTTG	55	This study
SIX6	SIX6 ALL F/R2	TATGCCTGAGCACACCATCAAT/ CTCCCAGAGCCATGTATA	50	This study
SIX6[2]	SIX6 FOP1 C481 F/R	AGCACATCAACGAGATCACG/ TTTGAAACACCAGGTATAGG	53	This study
SIX7	SIX7 FOL F/R	CATCTTTTCGCCGACTTGGT/ CTTAGCACCCTTGAGTAACT	59	Lievens et al. (2009)
SIX9	qSIX9 F/R	GCCGACCCAGACCTACGCTTT/ GCTGGTTTTGGAAGCCCAGTTGT	63	Taylor et al. (2019)
SIX10	FOC SIX10 F/R	GTTAGCAACTGCGAGACACTAGAA/ AGCAACTTCCTTCCTCTTACTAGC	63	Taylor et al. (2016)
SIX11	SIX11 FOP1 F/R	CGCAGAGGTTGACCAATAGGTC/ CCCAACTTGTTCTGGGGGATTT	61	This study
SIX12	SIX12 FOP1 F/R	GCCGTTCGCACTCCTAGTCATT/ CGCATCTCTTCCTTCGCGTACT	63	This study
SIX13	SIX13 FOP2/5 F/R	TCGAAATCCTTCATCATCGACAA/ TGTAGCGTTCAAACCACCCTTG	61	This study
SIX14	SIX14 FOL F/R	ATAAAGTGCGACTGGACTTCTGCC/ ACCCCCATCCACATTCCTAAGCGA	67	Taylor et al. (2016)
**Primers used for real time reverse transcription-polymerase chain reaction (qPCR)**				
TEF	qTEF F2/R2	GGTCAGGTCGGTGCTGGTTACG/ TGGATCTCGGCGAACTTGCAGG	63	Taylor et al. (2016)
SIX1	qSIX1 F3/R2	CGGTCTGTGCGTTGAAAGGTTTG/ CTCATTCTCCCCTCGGACATAG	63	This study
SIX6	qSIX6 ALL F3/R2	GAAGGCGAATTTATCATCTTTGG/ CTTTTCCCGGTTGCTGCGAG	62	This study
SIX7	qSIX7 F3/R3	TCGATCTCTTTCCAAGACAAGGGCA/ GTGGACGCGGCGTTGGTGAAC	63	Taylor et al. (2016)
SIX9	qSIX9 F/R	GCCGACCCAGACCTACGCTTT/ GCTGGTTTTGGAAGCCCAGTTGT	63	Taylor et al. (2019)
SIX10	qSIX10 F2/R2	CCCGGAAAGCCTGCATCGACTA/ AGAACAAACGTCGGTGGGACCA	63	Taylor et al. (2016)
SIX11	qSIX11 A F/R	GGCCACACCTGCACGAAAG/ CGCAGTTCTTCCCGTCTTTG	60	This study
SIX12	qSIX12 F3/R3	TGCTGCTCCAAGTACAAACTACCTT/ GCTGATACCTTTGGGTCCAACGC	63	Taylor et al. (2016)
SIX13	FON qSIX13 F2/R2	ACAGCACGGGACAGCTTACA/ CGTCAGAGGGGTAGCCACAT	60	Taylor et al. (2019)
SIX14	qSIX14 E F/R	GCTCTGTCTCAGCGTATCCTC/ CGACCTGAAACTACCGCCTG	62	This study

Table S4 Nucleotide substitution models used for *SIX* gene phylogenetic trees in MEGA7

**Gene**	**Model calculated and used**	**Reference**
SIX1	Tamura 3-parameter, gamma distributed	Tamura (1992)
SIX6	Kimura 2-parameter, gamma distributed	Kimura (1980)
SIX7	Kimura 2-parameter	Kimura (1980)
SIX9	Kimura 2-parameter	Kimura (1980)
SIX10	Kimura 2-parameter, gamma distributed	Kimura (1980)
SIX11	Jukes-Cantor	Jukes and Cantor (1969)
SIX12	Kimura 2-parameter	Kimura (1980)
SIX13	Jukes-Cantor, gamma distributed	Jukes and Cantor (1969)
SIX14	Kimura 2-parameter	Kimura (1980)

Table S5 Log$_e$ transformed ANOVA means of the expression of *Secreted In Xylem* (*SIX*) genes relative to the *translation elongation factor 1α* (*TEF*) gene for RNA extracted from pea roots infected with *Fusarium oxysporum* f. sp. *pisi* (FOP) isolates FOP1 EMR (race 1), F81 (race 2) and R2 (race 2^S) between 0 – 96 hpi. Significant differences between time points can be calculated using the 5% LSD[1], and between isolates for the same time point using the 5% LSD[2] (*SIX1*, *SIX6* and *SIX13* only).

Time (h)	Relative expression of *SIX* gene to *TEF* (transformed means)													
	1		***6***			***7***	***9***	***10***	***11***	***12***	***13***		***14***
	F81	R2	FOP1 EMR	F81	R2	FOP1 EMR	F81	FOP1 EMR	F1	FOP1 EMR	F81	R2	F81
0	-	-	-	-	-	-	-	-	-	-	-	-	-
8	-4.81	-5.67	-	~	~	~	-	~	-6.01	~	-7.56	-7.21	~
16	-3.94	-3.93	~	~	-6.83	~	~	~	-5.12	~	-7.66	-7.83	-5.89
24	-2.40	-2.31	=	-7.60	-5.85	-7.03	~	-8.95	-4.47	-7.88	-7.90	-7.98	-5.16
36	-0.71	-0.03	-7.52	-7.26	-4.64	-5.91	-8.27	-8.61	-3.17	-6.85	-8.04	-7.60	-4.75
48	-0.26	0.13	-6.72	-5.24	-4.01	-5.82	-7.05	-8.63	-3.48	-6.57	-7.10	-7.22	-4.25
72	-0.59	0.32	-6.01	-3.64	-3.78	-4.80	-6.32	-7.37	-2.89	-5.41	-6.80	-6.02	-4.34
96	-1.04	0.01	-5.53	-3.02	-3.57	-4.05	-6.17	-6.61	-2.01	-4.68	-5.39	-6.27	-4.35
LSD[1]	0.73		0.56			0.62	0.70	0.72	0.36	0.45	0.70		0.45
LSD[2]	0.61		0.42								0.70		

- Denotes missing values
~ Denotes average values containing missing values and those lower than the limit of detection of the primer pair
= Denotes all values below limit of detection for each primer pair

Table S6 Pathogenicity of six *Fusarium oxysporum* f. sp. *pisi* (FOP) isolates on four differential cultivars of pea (Little Marvel, DSP, Mini and Sundance II) 41 dpi. Data were transformed means for the number of wilted leaves as a proportion of the total number of leaves per pea plant following ANOVA analysis of logit transformed values for the root dip pathogenicity test.

Race	Isolate	Average proportion of wilted leaves (logit transformed)			
		**Little Marvel**	**DSP**	**Mini**	**Sundance II**
1	FOP1 EMR	2.75	-0.71	3.03	-3.26
	F79	2.42	-1.47	2.90	-3.65
2	FOP2	1.94	2.95	-2.41	-3.51
	F81	3.01	3.33	-0.90	-3.32
2^S	R2	2.55	3.27	-2.08	-3.53
	F40	2.93	3.34	0.11	-3.23
Non-inoculated	Control	-2.49	-2.52	-2.71	-3.23
	d.f				348
	5% LSD				1.10

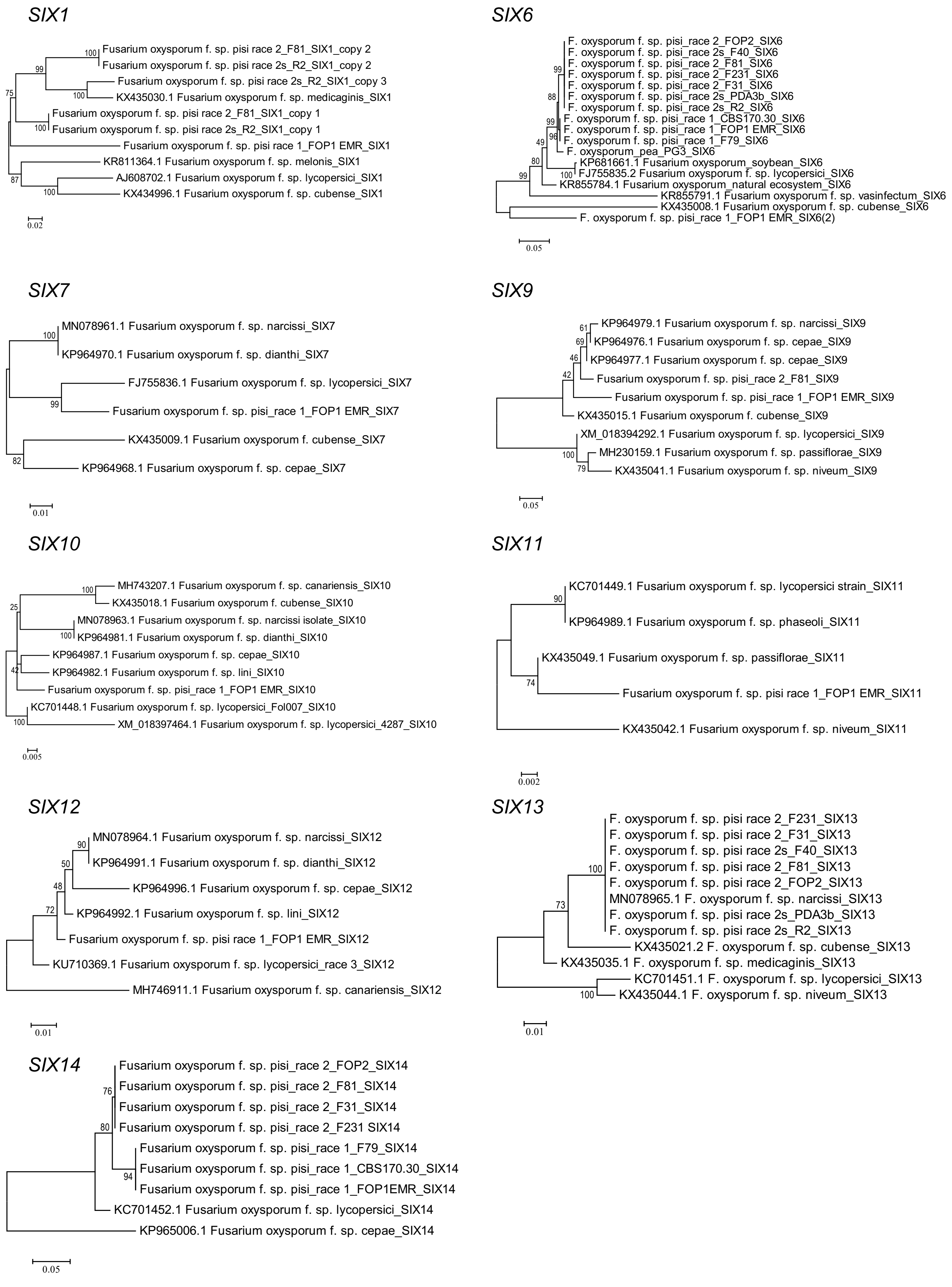

Figure S1 Maximum likelihood trees of *Fusarium oxysporum* isolates from pea and other hosts based on *Secreted In Xylem* (*SIX*) genes identified in *Fusarium oxysporum* f. sp. *pisi* isolates. Scale bars indicate the number of substitutions per site and numbers represent bootstrap percentage values from 1000 replicates.

Jukes, T.H., and Cantor, C.R. (1969). Evolution of protein molecules. *Mammalian protein metabolism* 3(21), 132.

Kimura, M. (1980). A simple method for estimating evolutionary rates of base substitutions through comparative studies of nucleotide sequences. *Journal of Molecular Evolution* 16(2), 111-120. doi: 10.1007/BF01731581.

Lievens, B., Houterman, P.M., and Rep, M. (2009). Effector gene screening allows unambiguous identification of Fusarium oxysporum f. sp. lycopersici races and discrimination from other formae speciales. *FEMS Microbiology Letters* 300(2), 201-215. doi: 10.1111/j.1574-6968.2009.01783.x.

Tamura, K. (1992). Estimation of the number of nucleotide substitutions when there are strong transition-transversion and G+C content biases. *Molecular Biology and Evolution* 9(4), 678-687.

Taylor, A., Armitage, A.D., Handy, C., Jackson, A.C., Hulin, M.T., Harrison, R.J., et al. (2019). Basal Rot of Narcissus: Understanding Pathogenicity in Fusarium oxysporum f. sp. narcissi. *Frontiers in Microbiology* 10(2905). doi: 10.3389/fmicb.2019.02905.

Taylor, A., Vagany, V., Jackson, A.C., Harrison, R.J., Rainoni, A., and Clarkson, J.P. (2016). Identification of pathogenicity-related genes in Fusarium oxysporum f. sp cepae. *Molecular Plant Pathology* 17(7), 1032-1047. doi: 10.1111/mpp.12346.
